# School lunchboxes as an opportunity for health and environmental considerations: a scoping review

**DOI:** 10.1093/heapro/daac201

**Published:** 2023-01-30

**Authors:** Neha Kishan Lalchandani, Brianna Poirier, Shona Crabb, Caroline Miller, Clare Hume

**Affiliations:** School of Public Health, University of Adelaide, Adelaide 5000, Australia; Australian Research Centre for Population Oral Health, Adelaide Dental School, University of Adelaide, Adelaide 5000, Australia; School of Public Health, University of Adelaide, Adelaide 5000, Australia; School of Public Health, University of Adelaide, Adelaide 5000, Australia; Health Policy Centre, South Australian Health and Medical Research Institute (SAHMRI), Adelaide 5000, Australia; School of Public Health, University of Adelaide, Adelaide 5000, Australia

**Keywords:** scoping review, school lunchboxes, child, nutrition, environment

## Abstract

Very little research has focussed on children’s school lunchboxes from both a health and environment standpoint. This scoping review explores studies that considered children’s lunchbox food consumption trends at school and the environmental impacts of lunchbox contents. We conducted a scoping review of peer-reviewed literature with a focus on lunchboxes of children in preschool or primary school settings that contained food packed from home, through the lens of food and nutrition in combination with environmental outcomes—particularly food and/or packaging waste. The review included 10 studies, with articles from Australia, USA, Spain, New Zealand and the UK. Half of them were intervention studies aiming to shift knowledge levels and attitudes of teachers, parents and children with regard to reducing packaged food choices and food waste, and improving dietary habits. Acknowledging the complexity of lunchbox packing and consumption practices, this review recommends the consideration of socio-ecological influences on children’s health and sustainability behaviour, and mobilizing their pro-environmental agency.

## INTRODUCTION

School food environments are critical to influencing children’s eating behaviours and childhood obesity (Driessen *et al.*, 2014; Welker *et al.*, 2016; Micha *et al.*, 2018). School food models vary globally, from school meal provision and canteen purchases to lunches packed from home. The latter model is common in Australia, where the current research was conducted, and is often compared with other approaches in the literature (Johnston *et al.*, 2012; Taylor *et al.*, 2019; Taher *et al.*, 2020). The literature is saturated with studies focussed on energy density measurements and nutritional quality assessments of children’s lunchboxes (Bell and Swinburn, 2004; Sanigorski *et al.*, 2005; Brennan *et al.*, 2010; Evans *et al.*, 2010; Sutherland *et al.*, 2020). These studies describe the commonality of energy-dense home-packed lunches containing foods high in fat, sodium and sugar, and low in fibre. The lack of fruits and vegetables in lunchboxes (Brennan *et al.*, 2010; Johnston *et al.*, 2012; Taylor *et al.*, 2019) and higher prevalence of discretionary foods and beverages (Bell and Swinburn, 2004; Sanigorski *et al.*, 2005; Sutherland *et al.*, 2020) is cause for concern from a health perspective. As a result, many interventions focus on increasing children’s consumptions of fruit and vegetables in preschools (Hodder *et al.*, 2017) and primary schools (Evans *et al.*, 2012), while simultaneously reducing intake of discretionary foods and sugar-sweetened beverages (SSBs) (Nathan *et al.*, 2019). Outcomes of school-based policies (Micha *et al.*, 2018) and interventions (Nathan *et al.*, 2019) to date have had mixed results, with mostly small to moderate effects lasting short term, with no significant impact on calorie intake or adiposity.

The importance of nutrition and nourishment for children’s health, academic performance, in-class focus and attentiveness (Taras, 2005; Burrows *et al.*, 2017), in combination with unsuccessful attempts to modify child eating behaviours, calls for innovative school-based strategies. One approach worthy of consideration is the marriage of environmental considerations with dietary behaviours to improve children’s health and environmental consciousness (Skouteris *et al.*, 2013; Friel *et al.*, 2014). Broader environmental impacts of school meals such as greenhouse gas emissions (GHGE) have been considered in the USA and some European countries (De Laurentiis *et al.*, 2017; Eustachio Colombo *et al.*, 2020; Poole *et al.*, 2020; Rossi *et al.*, 2021). Numerous studies have focussed on food or plate waste in school meal provision models to improve dietary intake and reduce food waste (Byker Shanks *et al.*, 2017; Metcalfe *et al.*, 2020; Kaur *et al.*, 2021). While environmental implications of ultra-processed foods are gaining prominence (Seferidi *et al.*, 2020), the child-proximal and potentially child-relevant outcomes of food waste and packaging waste from home-packed school lunches are yet to be investigated.

A recent review by O’Rourke *et al.* (O’Rourke *et al.*, 2020), which focussed on parental perceptions, experiences and habits with respect to home-packed school lunches, concluded that decisions influencing lunchbox packing behaviours are complex. Familial contexts and parental influence shape children’s dietary behaviours based on cultural, social and emotional norms (Savage *et al.*, 2007; Yee *et al.*, 2017), rather than the nutritional quality of food alone. Household income also influences access to high-quality healthy and unprocessed foods (French *et al.*, 2019). However, the presence of industrial or ultra-processed foods is becoming increasingly common in children’s lunchboxes as per recent reports (Nunes *et al.*, 2019; Barbosa *et al.*, 2021), regardless of socio-economic status.

Evidence has highlighted the importance of promoting behaviour change in children and adolescents, as habits developed in childhood are more likely to be sustained through adulthood (Kelder *et al.*, 1994; Lytle *et al.*, 2000). Hence, an environmental agenda could also be employed as an enabler of health and pro-environmental behaviours when parents or children themselves are packing school lunchboxes. The quality of lunchbox foods along with the packaging and waste outcomes is worth exploring in synergy as part of an interdisciplinary approach, as currently there are no explicit policies and programmes in school settings encompassing both aspects, despite plenty of latent activities existing already (Lalchandani et al., 2022). Therefore, this scoping review aimed to explore studies that considered both food present in children’s lunchboxes and the environmental impacts of lunchbox food contents. It focussed on children’s lunchboxes in preschool and primary school settings.

## METHODS

Systematic reviews are considered the highest level of evidence and often inform policy and practice (Munn *et al.*, 2018b). Scoping reviews, a sub-set of systematic reviews, are useful when determining the coverage of existing literature on a topic, particularly for emerging fields of inquiry (Arksey and O’Malley, 2005; Levac *et al.*, 2010; Munn *et al.*, 2018a). This scoping review was conducted to identify key characteristics of research that considers both children’s food consumption patterns and the environmental impacts of lunchbox foods. Considering these topics together is a new area of research, and hence we found conducting a scoping review useful to explore studies that encapsulate this overlap.

An initial search of PubMed, PROSPERO and the Joanna Briggs Systematic Reviews registry revealed no similar studies currently underway. In accordance with scoping review methodology (Peters *et al.*, 2020), the protocol was published with the Centre for Open Science (Foster and Deardorff, 2017) (https://osf.io) prior to the commencement of the systematic search (Lalchandani, 2022). This review was conducted and is reported in alignment with the Preferred Reporting Items for Systematic Reviews and Meta-Analyses (PRISMA) scoping review extension guidelines (Appendix 1).

### Information sources

Five databases were searched in October 2021 using index terms and keywords related to ‘children’, ‘preschool or primary/elementary school’, ‘lunchbox’, ‘food choice’ and ‘environment’ and ‘sustainability’. The search string was initially developed for PubMed and then adapted for each of EMBASE, SCOPUS, Web of Science and PsycINFO (Appendix 2). Literature published from database inception until October 2021 was considered for inclusion in this review. The search was not restricted by language or geographic location. After performing the search, all identified citations were collated and uploaded into Covidence (Veritas Health Innovation, Melbourne, Australia) and duplicates removed.

### Eligibility criteria and selection of sources

Two independent reviewers (N.K.L. and B.P.) conducted title and abstract screening, with articles considered potentially relevant by either reviewer advancing to full text review. Following full text retrieval, articles were independently screened by the reviewers against the predefined inclusion criteria:

- Children in preschool or primary school settings
- Food brought from home (alias packed lunches)
- Consideration of lunchbox nutrition or healthy eating in combination with environmental outcomes, food or packaging waste

The review team defined packed lunches as a lunch i.e. packed at home, either by parents or children themselves, and brought to school by the child to be consumed during snack or lunch break times. It is important to note that no federal regulations exist that instruct parents what can or cannot be packed, but there may be school-level policies that provide standards for packed lunches based on broader dietary guidelines available locally in their respective jurisdictions (Lucas *et al.*, 2017; Spence *et al.*, 2020).

Studies related to school meal provisions or canteen programmes were excluded. Any disagreements that arose during the screening processes were resolved through discussion or by a third reviewer (C.H.). The reference lists of all included studies were hand searched to identify any other relevant articles not captured by the systematic search.

### Data extraction and synthesis

Data were extracted into a piloted extraction form in Covidence by two reviewers (N.K.L. and B.P.). To ensure inter-reviewer reliability, extraction of three articles was performed by both reviewers. The data extracted included details about the study location, school type, study design, study aim, study methods, participants’ description, sample size, theoretical framework, definition of healthy food/healthy eating/healthy choices, definition of environmentally friendly/eco-friendly/sustainability, aspects of consideration (nutrition, food waste, packaging waste, broader environmental impacts), description of intervention (where applicable) and the main findings of the study. Extracted data were tabulated, categorically synthesized and narratively described. Interactions between child, parent and teacher stakeholders were synthesized considering the involvement of targeted populations in each study, along with study interventions and considerations discussed.

## RESULTS

**Fig. 1:**
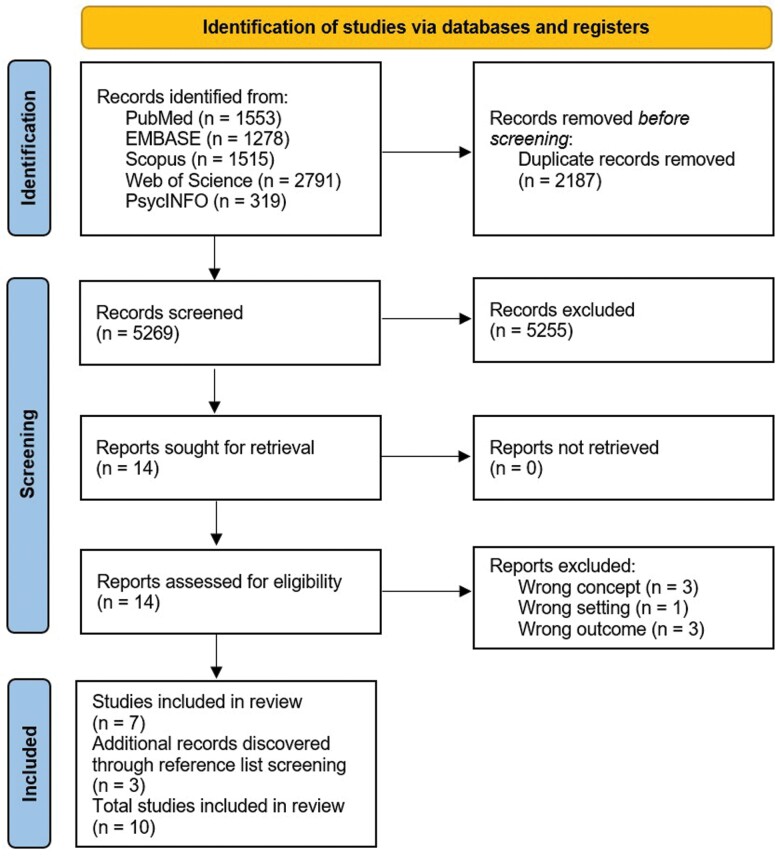
PRISMA diagram of the literature search process and article yield (Page *et al.*, 2021).

The systematic search identified 7456 studies, of which 2187 were duplicates, leaving 5269 unique records. During title and abstract screening, a further 5255 studies were excluded as they did not meet inclusion criteria, and the full text of 14 studies were then screened against inclusion and exclusion criteria. Seven studies satisfied the inclusion criteria and a further three studies were identified through reference searching; therefore, a total of 10 studies were included in this systematic scoping review (Figure 1).

Seven of the included studies took place in primary schools (Dresler-Hawke *et al.*, 2009; Goldberg *et al.*, 2015; Wickramasinghe *et al.*, 2016; Folta *et al.*, 2018; Boulet *et al.*, 2019; Antón-Peset *et al.*, 2021; Karpouzis *et al.*, 2021) and three of the studies were in preschools or early childhood centres (Edwards *et al.*, 2013; Boyd, 2015; Morris *et al.*, 2018). Authors of included studies utilized a range of methods to achieve their aims including direct observation for quantification of food waste or food packaging (Dresler-Hawke *et al.*, 2009; Goldberg *et al.*, 2015; Antón-Peset *et al.*, 2021), questionnaires (Morris *et al.*, 2018; Boulet *et al.*, 2019; Antón-Peset *et al.*, 2021; Karpouzis *et al.*, 2021), interviews (Boyd, 2015) and focus groups (Edwards *et al.*, 2013; Folta *et al.*, 2018; Morris *et al.*, 2018). Some of the included studies utilized a theoretical framework, including the socio-ecological model (Edwards *et al.*, 2013; Boyd, 2015), social cognitive theory (Goldberg *et al.*, 2015; Wickramasinghe *et al.*, 2016), the theory of reasoned action (Karpouzis *et al.*, 2021) and funds of knowledge (Morris *et al.*, 2018); others considered various approaches including behavioural (Boulet *et al.*, 2019) and social marketing practices (Folta *et al.*, 2018). Study characteristics based on extrapolation of data has been tabulated in Table 1 which also includes aims and main findings.

**Table 1:** Summary of identified studies focussing on health and environment aspects of school lunchboxes

First author name, year published, country (reference)	Study aim	Study design; methodology/intervention measures	Intervention design; duration	Participants	Main outcomes
Dresler-Hawke, 2009, New Zealand (Dresler-Hawke *et al.*, 2009)	To represent a snapshot of children’s food consumption behaviour at low- and high-socio-economic schools	Cross-sectional; direct observation that examined nutritional quality and food waste of lunchboxes	n/a	Primary school children aged 5–11 (lunchboxes *n* = 927)	Over 80% of unconsumed food items were sandwiches, fruit and dairy, compared with 20% that were energy-dense nutrient-poor snacks ‘junk food’
Edwards, 2013, Australia (Edwards *et al.*, 2013)	To develop a brief educational statement to support teachers in thinking about the relationship between children’s play, and curriculum with healthy eating, digital media/technology use and sustainability	Formative research; focus groups with children and parents to provide insights for subsequent teacher discussions	n/a	16 preschool children aged 4–5, 18 preparatory children aged 5–6, 34 mothers, and 6 preschool and primary school teachers	Influence of digital media and popular characters on children’s food preferences, the nutritional value of packaged food items and the sustainability issues associated with excess packaging of branded foods
Boyd, 2015, Australia (Boyd, 2015)	To investigate how educators implement healthy eating policies while promoting sustainable practices	Exploratory; qualitative interviews one-on-one with parents and teachers, and in small groups of three to four children	n/a	5 early childhood directors and 7 educators, 7 parents and 20 children	Contrasting perspectives of educators, parents and children is a barrier to healthy food choices and sustainable practices; a holistic approach is needed beyond the EC setting, improved educators’ knowledge and pedagogical practices, and empowerment of children to enact agency to be environmentally responsible
Goldberg, 2015, USA (Goldberg *et al.*, 2015)	To evaluate a communications campaign to motivate children to bring more fruits and vegetables and fewer SSBs to school	Cluster-randomized trial; direct observation that examined food and packaging	Multi-component, school-based intervention through classroom curriculum with variety of supplementary activities and parent communications; 7 months	582 primary school children in grades 3–4, mean age 9.1 years	Campaign was well received but no significant changes were observed in the quality of food brought to school and packaging type
Wickramasinghe, 2016, UK (Wickramasinghe *et al.*, 2016)	To quantify the nutritional quality and carbon footprint of school lunches and packed lunches	Retrospective cohort; quantification of GHGE of self-reported student lunchbox contents	n/a	Primary school children (lunchboxes *n* = 3488)	The mean GHGE of healthy packed lunches (0.39 kgCO_2_e) was lower than the mean GHGE of unhealthy packed lunches (0.72 kgCO_2_e)
Folta, 2018, USA (Folta *et al.*, 2018)	To develop a branding strategy to improve the quality of foods children bring from home to school, using a combined healthy eating and eco-friendly approach	Formative research; focus groups with parents and children	Two-phase branding strategy including development and testing of branding concepts; 4 months	73 primary school children in grades 3–4 and 17 parents	Environmental benefits of food choices were appealing for both parents and children, and they were receptive to the nutrition-eco concept through a brand that was simple, engaging, catered to various food preferences, and involved an element of mystery
Morris, 2018, Australia (Morris *et al.*, 2018)	To investigate the effect of teacher-designed play-based learning on children’s knowledge about wellbeing and sustainability	Randomized trial; questionnaires about eating and physical activity and qualitative analyses of visual art diaries and focus groups with children	Two professional learning sessions held with intervention group teachers, one session held with waitlist control teachers, supported with learning materials (Pedagogical Communication Strategy) and orientated to the concept of funds of knowledge to implement play-based learning experiences for preschool children; 8 weeks	25 early childhood teachers, 300 child–parent dyads	No increased knowledge connections immediately after intervention; but knowledge was sustained 3 months post intervention Intervention group ate more healthy foods and less packaged foods
Boulet, 2019, Australia (Boulet *et al.*, 2019)	To identify and prioritize food waste reduction behaviours	Exploratory case study; questionnaires for parents and children targeting behaviours related to food waste	n/a	110 primary school children aged 9–12 years and their parents (Note: high school children and parents not included in this review analysis)	Parents involved children in choosing, making and packing lunchbox food only sometimes or never; students only sometimes brought leftover food back home
Antón-Peset, 2021, Spain (Antón-Peset *et al.*, 2021)	To analyse whether a didactic intervention changes the level of knowledge and attitude towards food waste, and ultimately decreases quantity of food waste during mid-morning breaks (from home) and canteen lunches (not considered in this review context)	Single-case (embedded) design; questionnaires to analyse teacher and student knowledge, teaching and participatory activities and direct observation of food waste	Didactic intervention involving teaching sessions and activities, through active and participatory methodologies, and peer-based dissemination of information through posters to increase awareness and recognition of their role and responsibility as citizens; 3 months	One primary school teacher and 25 primary school children in grade 4 aged 9–10 years	Subtle changes in the level of knowledge and attitude towards food waste; decrease of almost half of the average weight (kg) of food waste per day in the rest of the primary school students’ cohort
Karpouzis, 2021, Australia (Karpouzis *et al.*, 2021)	To report the protocol for impact and process evaluation of a school-based FEAST programme	Parallel, cluster non-randomized controlled trial; curriculum delivery, online questionnaire with quantitative and qualitative components for school administrators	FEAST—ecological intervention through curriculum-aligned classroom education and cooking activities facilitated by teachers, parents, community volunteers; 10 weeks	20 primary schools (10 intervention vs. 10 wait-list-control); children in grades 5–6 aged 10–12 years	Results from this trial will provide valuable information on the value of adding environmental sustainability strategies to nutrition education in schools

### Synthesis of evidence

This scoping review mapped literature in the area of the environmental impacts of school lunchbox food, particularly the more immediate food and packaging waste attributes. The synthesis of evidence is described below, relating to the definitions used in the included studies, as well as the stakeholder interactions and outcomes of both intervention and observational studies.

#### Definitions

Three varying definitions relating to content of lunchboxes were used across the included studies: (i) Natural/whole/unprocessed vs. packaged/junk/processed foods (Boyd, 2015; Goldberg *et al.*, 2015; Folta *et al.*, 2018; Morris *et al.*, 2018; Karpouzis *et al.*, 2021); (ii) Food choices that follow advice based on guidelines and policies (Dresler-Hawke *et al.*, 2009; Boyd, 2015); (iii) Nutrient-rich vs. nutrient-poor quality of lunchbox foods (mainly saturated fats, salt and sugar) (Dresler-Hawke *et al.*, 2009; Edwards *et al.*, 2013; Goldberg *et al.*, 2015; Wickramasinghe *et al.*, 2016; Folta *et al.*, 2018). Two studies (Boulet *et al.*, 2019; Antón-Peset *et al.*, 2021) had neither an explicit definition or an indirect reference to one for healthy foods or healthy eating as they were food waste focussed. As a result of definitions employed, comparisons were often made between whole foods such as fruits, vegetables, whole grains, water and junk foods such as discretionary snacks, SSBs, confectionery and desserts. Similarly, included studies defined environmental or sustainable aspects based on three characteristics: (i) Reducing or avoiding food waste (Boulet *et al.*, 2019; Antón-Peset *et al.*, 2021; Karpouzis *et al.*, 2021); (ii) Environmental impacts of excess packaging and highly processed foods (Edwards *et al.*, 2013; Boyd, 2015; Goldberg *et al.*, 2015; Folta *et al.*, 2018; Morris *et al.*, 2018); (iii) Greenhouse gas emissions (Wickramasinghe *et al.*, 2016). One study did not have a definition (or indirect reference to one) for environmental or sustainable considerations even though it was food waste focussed (Dresler-Hawke *et al.*, 2009).

#### Intervention studies: stakeholder interactions

Five of the included studies described interventions (Goldberg *et al.*, 2015; Folta *et al.*, 2018; Morris *et al.*, 2018; Antón-Peset *et al.*, 2021; Karpouzis *et al.*, 2021), which largely focussed on increasing healthy food consumption while decreasing packaged foods (Goldberg *et al.*, 2015; Folta *et al.*, 2018; Morris *et al.*, 2018), as well as food waste awareness (Karpouzis *et al.*, 2021) and reduction (Antón-Peset *et al.*, 2021). The nature of the interventions varied across three primary stakeholder groups—teachers, parents and children.

Teachers were provided training and informative resources (Goldberg *et al.*, 2015; Morris *et al.*, 2018; Antón-Peset *et al.*, 2021; Karpouzis *et al.*, 2021), and this allowed for knowledge transfer to children via curriculum and inquiry-based learning (Goldberg *et al.*, 2015; Antón-Peset *et al.*, 2021; Karpouzis *et al.*, 2021), play-based learning (Morris *et al.*, 2018) and experiential activities such as cooking (Karpouzis *et al.*, 2021). Interventions that were integrated into school lessons aimed to teach children actionable ways to packing and consuming healthy foods (Goldberg *et al.*, 2015; Morris *et al.*, 2018), increase food waste awareness and knowledge (Antón-Peset *et al.*, 2021; Karpouzis *et al.*, 2021) and improve food literacy in context of nutrition, food preparation and cooking (Karpouzis *et al.*, 2021). Two studies had poster creation activities for children: one aimed to raise food waste awareness via peer-to-peer cascade learning process (Antón-Peset *et al.*, 2021) and the other sought to capture a campaign’s impact on students across the school (Goldberg *et al.*, 2015). The latter provided campaign information via parent-teaching meetings and other school events, however knowledge transfer in this project was expected to occur via children who relayed their food requests to parents at home (Goldberg *et al.*, 2015; Antón-Peset *et al.*, 2021). Parents and children were also directly involved in another study that aimed to gauge their receptiveness to a nutrition-eco campaign (Folta *et al.*, 2018).

#### Intervention studies: outcomes

Intervention outcomes largely focussed on decreased food waste and increased consumption of healthy foods, although none of the outcomes were the same across the included studies. However, few comparisons can be made across the five studies. Antón-Peset’s multi-component intervention based in Spain (Antón-Peset *et al.*, 2021) was 3 months in duration and resulted in a decrease in food waste from mid-morning break snacks by almost half in the group of students not exposed to the intervention directly. This was a result of the didactic intervention sequence and peer-learning process whereby intervention group students showed and explained the informative food waste themed posters to their peers. By contrast, Goldberg *et al.*’s American school-based nutrition-eco communications campaign called Great Taste, Less Waste (Goldberg *et al.*, 2015) which lasted 7 months and aimed to increase fruit and vegetable content in lunchboxes and reduce SSBs along with single-serve packaged food items, resulted in negligible changes in the quality of lunches and packaging reduction. Subsequently, a 4-month formative research study by Folta *et al.* also based in America (Folta *et al.*, 2018) had more favourable attributes, highlighting the importance of simple intervention designs and the direct involvement of children and parents in campaign development. Similarly, another study (Antón-Peset *et al.*, 2021) acknowledged the advantages of directly targeting children in interventions rather than relying on knowledge transfer to children by teachers and parents.

Findings from Morris *et al.* (Morris *et al.*, 2018), 3 months post an 8-week intervention, demonstrated a significantly higher knowledge connection between health and the environment, and children ate more healthy foods and less packaged foods among the intervention group. Their findings suggest moving away from the health promotion approach i.e. top-down in nature and instead encourage a shift towards a ground-up approach connecting play-based learning experience with health and sustainability knowledge (Morris *et al.*, 2018). Although the Australian OZHarvest Food Education and Sustainability Training (FEAST) programme study was a protocol for a 10-week intervention and did not report any trial outcomes (at the time of this review) (Karpouzis *et al.*, 2021), building children’s skills and capabilities alongside their knowledge were shared recommendations from Morris *et al.* (Morris *et al.*, 2018).

#### Observational studies: stakeholder interactions

Five of the included studies were not interventions (Dresler-Hawke *et al.*, 2009; Edwards *et al.*, 2013; Boyd, 2015; Wickramasinghe *et al.*, 2016; Boulet *et al.*, 2019); two focussed on food waste (Dresler-Hawke *et al.*, 2009; Boulet *et al.*, 2019), one considered the broader environmental impact of school lunchboxes by measuring GHGE (Wickramasinghe *et al.*, 2016), and two were exploratory studies that examined the overlap between healthy eating and environment (Edwards *et al.*, 2013; Boyd, 2015).

Two Australian studies considered stakeholders in all three categories: in one of these studies, parent and children perspectives were shared with educators to help develop educational statements (Edwards *et al.*, 2013) and the other study considered all perspectives concurrently (Boyd, 2015). Despite these differences, both studies had similar findings. At the parent level, food choices and sustainability practices varied widely from the school’s healthy food policies. Both research groups identified the importance of increasing educator capacity and providing support to encourage teachers to navigate their role towards children’s health and wellbeing, respecting and valuing parents’ food choices for their children, and understanding the social and cultural aspects of environments beyond school settings. At the school level, the importance of embedding food and sustainability connections in the curriculum and pedagogical practices was described as central to enabling children to enact agency, develop social responsibility and pave the path to healthy and sustainable eating practices (Koch, 2016).

#### Observational studies: outcomes

The five studies that were not intervention based had shared considerations of lunchbox nutrition quality and environmental outputs. Both Boulet *et al.* (Boulet *et al.*, 2019) and Dresler-Hawke *et al.* (Dresler-Hawke *et al.*, 2009) had a food waste focus and to reduce it suggested solutions that relied on modification of school environments, such as restructuring timetables to increase eating time or scheduling eating time after play time. They also recommended curriculum-based educational reforms to fulfil health and environmental agendas and develop children’s self-efficacy in school. Dresler-Hawke *et al.* went further and advocated for partnerships between school and home environments to increase parental awareness of children’s food eating and waste behaviours (Dresler-Hawke *et al.*, 2009). Dissimilar to other studies included in this review, Wickramasinghe’s study (Wickramasinghe *et al.*, 2016) considered nutritional aspects of lunchbox food in terms of nutrient and micronutrient content and associated GHGE of lunchbox items in England. The findings of this study were conflicting due to the complexity of defining healthy and unhealthy packed lunches; e.g. when accounting for micronutrients (iron, calcium, zinc and folate) the GHGE of healthy packed lunches was larger than unhealthy lunches but when accounting for salt, fat and sugar, the GHGE of unhealthy packed lunches was larger.

## DISCUSSION

This scoping review explored existing literature that considered school children’s lunchbox contents from both health and environmental perspectives. A total of 10 articles were included and half of them were intervention studies, intending to change behaviour via knowledge levels and attitudes of teachers, parents and children with regard to healthy eating and sustainability practices. Although four of five interventions discussed in this review were between 2 and 4 months in duration, Goldberg *et al.*’s intervention which was the longest in duration (7 months) and also the most complex did not work as well. There was more inclination towards simpler interventions through active participatory approaches, and motivating children to recognize their role and responsibility to be drivers of change in the environmental landscape.

Metcalfe *et al.* very aptly described the lunchbox as ‘*a space or “container” into which various aspects of the school and the home—the public and the private—may be packed*’ (Metcalfe *et al.*, 2008). This was also reflected in interventions discussed in this review encompassing complex and multifaceted pathways involving teacher training, raising children’s awareness, knowledge and skills, and influencing parent decision making. In particular, Morris *et al.* (Morris *et al.*, 2018) highlighted the importance of active educator and parental involvement in children’s health outcomes and the influence of children’s home life on their food choices and sustainability behaviours. Holistic educational approaches encompassing all stakeholders and moving beyond silo approaches were deemed necessary by two studies included in this review (Edwards *et al.*, 2013; Boyd, 2015). This is especially relevant when recognizing the relationships between individuals and the ever-changing environments of multi-level systems (Bronfenbrenner, 1992).

Although lunchboxes draw the involvement of children, parents, educators and the broader socio-ecological systems within which the aforementioned stakeholders are embedded, there is merit for the argument that interventions should directly target children and turn away from regulating their food choices during school time. Lunchbox surveillance by teachers has transformed into supposed pedagogical opportunities, mainly through the way they respond or react to certain lunchbox contents often hinting towards judgement (Pluim *et al.*, 2018). Two of the studies in this review also shed light on the tensions between educators and parents as both parties have differing perspectives and priorities (Edwards *et al.*, 2013; Boyd, 2015). Reliance on parental involvement in interventions is not without its own set of complications, sensitivities and concerns (Edwards *et al.*, 2013; Boyd, 2015; Folta *et al.*, 2018; Boulet *et al.*, 2019; O’Rourke *et al.*, 2020). There may also be food accessibility and availability differences across households as differing circumstances and financial abilities would impact what parents can or cannot provide as food in lunchboxes. Although food insecurity is likely to be an important influence on children’s school lunchbox contents, several of the studies reviewed performed direct observation of lunchboxes only; thus, there was no opportunity to gather or account for data such as food security. Household income, while a good indicator of individual-level socio-economic status, is not necessarily a good proxy for food security either (Kleve *et al.*, 2018). Therefore, future research can explore how household food security influences what’s packed in lunchboxes. Moreover, given this review focussed on studies examining school lunchboxes, it is not surprising that most studies took educative approaches, either around healthy food, or skill-based studies such as cooking and food preparation. Studies examining the use of income supports and the effects on school lunchboxes would be a very interesting avenue for future research as this appears to be currently understudied.

The integral role that parents play in children’s lives and their food consumption behaviours cannot be understated as they remain ‘gatekeepers’. However, children’s preferences and food requests often take greater precedence regardless of socio-economic positions (Johnson *et al.*, 2020) and hence, future interventions could target children to increase their food literacy. Particularly, it would be worthwhile to focus on foods as whole and pragmatically linking those choices to environmental impacts, as Ronto *et al.*’s study findings showed that adolescents had limited knowledge connecting food consumption with environmental sustainability (Ronto *et al.*, 2016).

This review, among other literature, highlights the power of children’s voices in making food requests before the lunchbox is packed and then making food decisions within the lunchbox itself after it is packed (Bathgate and Begley, 2011; Ensaff *et al.*, 2018). Creating child-focussed interventions aligns with the United Nations Convention on the Rights of the Child and the ‘new’ sociology of childhood (UNICEF, 1989) that honours the autonomy and power that children hold as agents of change (James, 2010). Young children have demonstrated the capability to internalize complex environmental issues and this awareness has the potential to motivate children to make ‘healthier’ and sustainable food choices (Cutter-Mackenzie, 2010; Skouteris *et al.*, 2013; Kos *et al.*, 2016); this phenomenon was evident in three of the primary year level interventions discussed in this review (Goldberg *et al.*, 2015; Folta *et al.*, 2018; Antón-Peset *et al.*, 2021). Hence, we propose future interventions focus on developing children’s self-efficacy and encourage their active participation and involvement as agents of change. Conducting formative and exploratory research is necessary to better understand the perceptions and requirements of this target group and will make desired intervention outcomes more achievable (Folta *et al.*, 2018; Morris *et al.*, 2018; Karpouzis *et al.*, 2021).

The variation in definitions of healthy eating employed across the included studies reflects the purely ‘conceptual simplicity’ (Neufeld *et al.*, 2021) of nutritious foods. Understandings of nutrition and ‘healthy’ foods are dependent on specific contexts, which also means that characterization of healthy diets is influenced by a range of external determinants. While some of the definitions converged with broader definitions utilized by the United Nations (Neufeld *et al.*, 2021) and World Health Organization (World Health Organization, 2019), there is no formal or universal guide for school lunchbox contents. Moreover, environmental agendas and behavioural priorities are not uniform across schools; however, the Sustainable Development Goals (Resolution, 2015) in areas of education, health and wellbeing and environment underpinned the development of one intervention included in this review (Antón-Peset *et al.*, 2021). Similarly, Australian based interventions were embedded into curriculum via pre-existing National Quality Standard and Early Years Learning Framework (Boyd, 2015; Morris *et al.*, 2018; Karpouzis *et al.*, 2021). We recommend aligning intervention aims and objectives in future programming with international policies and guidelines to ensure relevance and garner international support and understanding of interventions. The development of a realistic and achievable health definition specifically for lunchbox foods that also considers sustainability would be transformative for this area of research.

### Strengths and limitations

To our knowledge, this is the first review to consider programmes incorporating an environmental focus when reviewing lunchbox studies alongside health and nutrition characteristics. Given this novel and emerging area of research, this scoping review provides a basis for future work in this field. Additionally, this review was conducted in alignment with the PRISMA extension for scoping reviews and a protocol was published and made publicly available prior to conducting the review. The robust method involved searching a range of databases and two researchers who reviewed the included and excluded studies. This review was limited to peer-reviewed articles in English and as a result some studies may have been omitted that were published in different languages. Despite best efforts to include all relevant terminologies pertinent to the research question, due to the variation in definitions and terms for both healthy foods and environmental considerations, some studies eligible for inclusion may not have been captured by the search strategy.

## CONCLUSIONS

This review provides insights relevant for school food settings that rely on a packed lunch from home model. Lunchbox packing and consumption is complex, and it involves input from various sources. Even though the various stakeholders involved are not always working towards the same goal, a handful of studies showed intervention successes and even those without significant changes provided useful recommendations for future interventions. Future efforts that consider both the food and environmental aspects of packed lunchboxes should consider the socio-ecological influences on children’s health and sustainability behaviour. Schools can consider changing their food settings so they can be more conducive to children’s healthy and sustainable eating patterns. Teachers can integrate synergistic ideas that combine nutrition and sustainability into their curriculum. Parents can be supported by schools and policies to provide children with nutritious and environmentally friendly foods when packing lunchboxes. Children have the power to request foods based on their preferences, and often make choices before and after their lunchboxes are packed. In line with the studies reviewed in this article, there was a strong consideration of children’s agency, and we recommend mobilizing this avenue to drive behaviour change for their health and environmental sustainability.

## Supplementary Material

daac201_suppl_Supplementary_Appendix_1

daac201_suppl_Supplementary_Appendix_2
