# Supplementary material for: School lunchboxes as an opportunity for health and environmental considerations: a scoping review
Source: Health Promot Int. 2023 Jan 30;38(1):daac201. doi: 10.1093/heapro/daac201 (PMC9885980; doi:10.1093/heapro/daac201)
Supplement: daac201_suppl_Supplementary_Appendix_2 [file daac201_suppl_supplementary_appendix_2.docx]

**SEARCH STRATEGY FOR**

**School lunchboxes as an opportunity for health and environmental considerations: A Scoping Review**

**Logic Grid: PubMed**

[https://pubmed.ncbi.nlm.nih.gov/advanced/](https://pubmed.ncbi.nlm.nih.gov/advanced/\)

| Setting | Behaviour (Healthy Food Choice) | Environmental Factors |
| --- | --- | --- |
| “child”[mh] OR “schools, nursery”[mh] OR “schools”[mh:noexp] OR child*[tw] OR kid[tw] OR kids[tw] OR school*[tw] OR preschool[tw] OR kindergarten[tw] OR “school health services”[mh:noexp] OR lunchbox*[tw] OR elementary school*[tw] | “food and beverages”[mh] OR “diet”[mh] OR food consumption behavio?r*[tw] OR healthy diet*[tw] OR healthy food choice*[tw] OR “nutritional physiological phenomena”[mh] OR food decision*[tw] OR food decision making[tw] OR food choice*[tw] OR “food preferences”[mh] OR food preference*[tw] OR food choice motives[tw] OR ((“self control”[mh] OR “personal autonomy”[mh]) AND (food*[tw] OR diet*[tw])) OR fruit intake[tw] OR vegetable intake[tw] OR school nutrition[tw] OR nutrition* education[tw] | sustainability[tw] OR environmentally friendly[tw] OR eco-friendly[tw] OR “eco friendly”[tw] OR ecofriendly[tw] OR pro-environmental[tw] OR environmental stewardship[tw] OR environmentally conscious[tw] OR environmental sustainability[tw] OR planetary health[tw] OR environmental impact*[tw] OR “environmental policy”[mh] OR school garden*[tw] OR ((“environment”[mh] OR “climate change”[mh]) AND “health”[mh]) OR ecological footprint[tw] OR greenhouse gas emission[tw] OR carbon footprint[tw] OR ((food[tw] OR diet[tw]) AND climate change[tw]) OR ((food[tw] OR diet[tw]) AND global warming[tw]) OR food waste[tw] OR environment* education[tw] |

**Logic Grid: EMBASE**

<https://www.embase.com/#advancedSearch/default>

| Setting | Behaviour (Healthy Food Choice) | Environmental Factors |
| --- | --- | --- |
| child/de OR “preschool child”/de OR “school child”/de OR school/de OR child*:ti,ab,kw OR kid:ti,ab,kw OR kids:ti,ab,kw OR school*:ti,ab,kw OR preschool:ti,ab,kw OR kindergarten:ti,ab,kw OR “school health service”:ti,ab,kw OR “lunchbox*”:ti,ab,kw OR “elementary school*”:ti,ab,kw | food/de OR diet/de OR nutrition/de OR “food consumption behavio?r*”:ti,ab,kw OR “healthy diet*”:ti,ab,kw OR “healthy food choice*”:ti,ab,kw OR “food decision*”:ti,ab,kw OR “food decision making”:ti,ab,kw OR “food choice*”:ti,ab,kw OR “food preference”/de OR “food preference*”:ti,ab,kw OR “food choice motives”:ti,ab,kw OR ((“self control”/de OR “personal autonomy”/de) AND (food*:ti,ab,kw OR diet*:ti,ab,kw)) OR “fruit intake”:ti,ab,kw OR “vegetable intake”:ti,ab,kw OR “school nutrition”:ti,ab,kw OR “nutrition* education”:ti,ab,kw | sustainability:ti,ab,kw OR “environmentally friendly”:ti,ab,kw OR eco-friendly:ti,ab,kw OR ecofriendly:ti,ab,kw OR pro-environmental:ti,ab,kw OR “environmental stewardship”:ti,ab,kw OR “environmentally conscious”:ti,ab,kw OR “environmental sustainability”:ti,ab,kw OR “planetary health”:ti,ab,kw OR “environmental impact*”:ti,ab,kw OR “environmental policy”/de OR “school garden*”:ti,ab,kw OR ((environment/de OR “climate change”/de) AND health/de) OR “ecological footprint”:ti,ab,kw OR “greenhouse gas emission”:ti,ab,kw OR “carbon footprint”:ti,ab,kw OR ((food:ti,ab,kw OR diet:ti,ab,kw) AND “climate change”:ti,ab,kw) OR ((food:ti,ab,kw OR diet:ti,ab,kw) AND “global warming”:ti,ab,kw) OR “food waste”:ti,ab,kw OR “environment* education”:ti,ab,kw |

**Logic Grid: SCOPUS**

<https://www.scopus.com/search/form.uri?display=basic&clear=t&origin=searchadvanced&txGid=5bbd2936ad87e3c26bec5bb6cfb22c32#basic>

| Setting | Behaviour (Healthy Food Choice) | Environmental Factors |
| --- | --- | --- |
| child OR “preschool child” OR “school child” OR child* OR kid OR kids OR school OR school* OR preschool OR kindergarten OR “school health service” OR lunchbox* OR “elementary school*” | food OR diet OR nutrition OR “food consumption behavio?r*” OR “healthy diet*” OR “healthy food choice*” OR “food decision*” OR “food decision making” OR “food choice*” OR “food preference” OR “food preference*” OR “food choice motives” OR ((“self control” OR “personal autonomy”) AND (food* OR diet*)) OR “fruit intake” OR “vegetable intake” OR “school nutrition” OR “nutrition* education” | sustainability OR “environmentally friendly” OR eco-friendly OR eco friendly OR ecofriendly OR pro-environmental OR “environmental stewardship” OR “environmentally conscious” OR “environmental sustainability” OR “planetary health” OR “environmental impact*” OR “environmental policy” OR “school garden*” OR ((environment OR “climate change”) AND health) OR “ecological footprint” OR “greenhouse gas emission” OR “carbon footprint” OR ((food OR diet) AND “climate change”) OR ((food OR diet) AND “global warming”) OR “food waste” OR “environment*al education” |

**Logic Grid: Web of Science**

<https://www.webofscience.com/wos/woscc/summary/5c28328d-834d-4395-ae92-386ba8b04f71-00dc4be9/relevance/1>

| Setting | Behaviour (Healthy Food Choice) | Environmental Factors |
| --- | --- | --- |
| TI=(child OR “preschool child” OR “school child” OR child* OR kid OR kids OR school OR school* OR preschool OR kindergarten OR “school health service”) OR AB=(child OR “preschool child” OR “school child” OR child* OR kid OR kids OR school OR school* OR preschool OR kindergarten OR “school health service”) OR AK=(child OR “preschool child” OR “school child” OR child* OR kid OR kids OR school OR school* OR preschool OR kindergarten OR “school health service” OR “lunchbox*” OR “elementary school*” ) | TI=(food OR diet OR nutrition OR “food consumption behavio?r*” OR “healthy diet*” OR “healthy food choice*” OR nutrition OR “food decision*” OR “food decision making” OR “food choice*” OR “food preference” OR “food preference*” OR “food choice motives” OR ((“self control” OR “personal autonomy”) AND (food* OR diet*)) OR “fruit intake” OR “vegetable intake” OR “school nutrition” OR “nutrition education” ) OR AB=(food OR diet OR nutrition OR “food consumption behavio?r*” OR “healthy diet*” OR “healthy food choice*” OR nutrition OR “food decision*” OR “food decision making” OR “food choice*” OR “food preference” OR “food preference*” OR “food choice motives” OR ((“self control” OR “personal autonomy”) AND (food* OR diet*)) OR “fruit intake” OR “vegetable intake” OR “school nutrition” OR “nutrition education” ) OR AK=(food OR diet OR nutrition OR “food consumption behavio?r*” OR “healthy diet*” OR “healthy food choice*” OR nutrition OR “food decision*” OR “food decision making” OR “food choice*” OR “food preference” OR “food preference*” OR “food choice motives” OR ((“self control” OR “personal autonomy”) AND (food* OR diet*)) OR “fruit intake” OR “vegetable intake” OR “school nutrition” OR “nutrition education” ) | TI=(sustainability OR “environmentally friendly” OR eco-friendly OR eco friendly OR ecofriendly OR pro-environmental OR “environmental stewardship” OR “environmentally conscious” OR “environmental sustainability” OR “planetary health” OR “environmental impact*” OR “environmental policy” OR “school garden*” OR ((environment OR “climate change”) AND health) OR “ecological footprint” OR “greenhouse gas emission” OR “carbon footprint” OR ((food OR diet) AND “climate change”) OR ((food OR diet) AND “global warming”) OR “food waste” OR “environmental education” ) OR AB=(sustainability OR “environmentally friendly” OR eco-friendly OR eco friendly OR ecofriendly OR pro-environmental OR “environmental stewardship” OR “environmentally conscious” OR “environmental sustainability” OR “planetary health” OR “environmental impact*” OR “environmental policy” OR “school garden*” OR ((environment OR “climate change”) AND health) OR “ecological footprint” OR “greenhouse gas emission” OR “carbon footprint” OR ((food OR diet) AND “climate change”) OR ((food OR diet) AND “global warming”) OR “food waste” OR “environmental education” ) OR AK=(sustainability OR “environmentally friendly” OR eco-friendly OR eco friendly OR ecofriendly OR pro-environmental OR “environmental stewardship” OR “environmentally conscious” OR “environmental sustainability” OR “planetary health” OR “environmental impact*” OR “environmental policy” OR “school garden*” OR ((environment OR “climate change”) AND health) OR “ecological footprint” OR “greenhouse gas emission” OR “carbon footprint” OR ((food OR diet) AND “climate change”) OR ((food OR diet) AND “global warming”) OR “food waste” OR “environmental education” ) |

**Logic Grid: PsycINFO**

<https://ovidsp.ovid.com/ovidweb.cgi?T=JS&NEWS=N&PAGE=main&SHAREDSEARCHID=333W2EIW6YObX4qY66vwhoLR0LT6fdOc5LjzudfDhyL9ceLIUvdejDaIjgmXR2jmx>

| Setting | Behaviour (Healthy Food Choice) | Environmental Factors |
| --- | --- | --- |
| child.sh OR preschool students.sh OR primary school students.sh OR School Based Intervention.sh OR school.sh OR child*.ti,ab OR kid.ti,ab OR kids.ti,ab OR school*.ti,ab OR preschool.ti,ab OR kindergarten.ti,ab OR school health service.ti,ab OR lunchbox*.ti,ab OR elementary school.ti,ab | food.sh OR diet.sh OR nutrition.sh OR food consumption behavio?r*.ti,ab OR healthy diet*.ti,ab OR healthy food choice*.ti,ab OR food decision*.ti,ab OR food decision making.ti,ab OR food choice*.ti,ab OR food preferences.sh OR food preference*.ti,ab OR food choice motives.ti,ab OR ((self control.sh OR autonomy.sh) AND (food*.ti,ab OR diet*.ti,ab)) OR fruit intake.ti,ab OR vegetable intake.ti,ab OR school nutrition.ti,ab OR nutrition* education.ti,ab | Sustainability.ti,ab OR environmentally friendly.ti,ab OR eco-friendly.ti,ab OR ecofriendly.ti,ab OR pro-environmental.ti,ab OR environmental stewardship.ti,ab OR environmentally conscious.ti,ab OR environmental sustainability.ti,ab OR planetary health.ti,ab OR environmental impact*.ti,ab OR environmental policy.sh OR school environment.sh OR school garden*.ti,ab OR ((environment.sh OR climate change.sh) AND health.sh) OR ecological footprint.ti,ab OR greenhouse gas emission.ti,ab OR carbon footprint.ti,ab OR ((food.ti,ab OR diet.ti,ab) AND climate change.ti,ab) OR ((food.ti,ab OR diet.ti,ab) AND global warming.ti,ab) OR food waste.ti,ab OR environment* education.ti,ab |
